# The relation of mTOR with diabetic complications and insulin resistance in patients with type 2 diabetes mellitus

**DOI:** 10.1186/s13098-024-01450-5

**Published:** 2024-09-11

**Authors:** Noha G. Amin, A. Abdel Rahim, Kamel Rohoma, Reham A.Abo Elwafa, Hossam M. F. Dabees, Shimaa Elrahmany

**Affiliations:** 1 Department of Internal Medicine (Diabetes, Lipidology & Metabolism), Faculty of Medicine, Alexandria University, 17, Champollion Street, El Messallah, Alexandria, Egypt; 2 Department of Clinical and Chemical Pathology, Faculty of Medicine, Alexandria University, Alexandria, Egypt

**Keywords:** Type 2 diabetes, mTOR, Insulin resistance, Microvascular complications

## Abstract

**Background:**

Dysregulation of the mechanistic target of rapamycin (mTOR) has been related to several metabolic conditions, notably obesity and type 2 diabetes (T2DM). This study aimed to evaluate the role of mTOR in patients with T2DM, and its relationship with insulin resistance and microvascular complications.

**Methods:**

This case-control study was conducted on 90 subjects attending the Outpatient Internal Medicine Clinic in Damanhur Teaching Hospital. Subjects were divided into 3 groups, Group I: 20 healthy controls, Group II: 20 subjects with T2DM without complications, and Group III: 50 subjects with T2DM with microvascular complications. An Enzyme-linked immunosorbent assay was used to measure serum mTOR levels. T2DM and diabetic complications were defined according to the diagnostic criteria of the American Diabetes Association.

**Results:**

The results revealed significant positive correlations to HbA1c (*r* = 0.530, *P* < 0.001), fasting glucose (*r* = 0.508, *P* < 0.001), and HOMA- IR (*r* = 0.559, *P* < 0.001), and a significant negative correlation to eGFR (*r*=-0.370, *P* = 0.002). Multivariate analysis revealed an independent association of mTOR and HbA1c values with the presence of microvascular complications. The prediction of microvascular complications was present at a cutoff value of 8 ng/ml mTOR with a sensitivity of 100% and specificity of 95% with an AUC of 0.983 and a *p*-value < 0.001.

**Conclusion:**

mTOR is a prognostic marker of diabetic microvascular and is associated with insulin resistance in patients with T2DM.

**Trial Registration:**

The study was conducted following the Declaration of Helsinki, and approved by the Ethics Committee of Alexandria University (0201127, 19/7/2018).

## Introduction

Diabetes mellitus (DM) is counted among the oldest diseases recognized by humans; it was first described by ancient Egyptian physicians 3500 years ago [1]. Currently, DM is deemed one of the most common chronic diseases worldwide, and its prevalence is increasing. Moreover, in addition to being a major consumer of global health expenditure, DM is a main driver of morbidity and mortality [2, 3].

Hyperglycemia, the hallmark of DM, induces microvascular damage through several mechanisms, such as polyol pathway flux, increased advanced glycation end products (AGEs) synthesis, persistent protein kinase C activation and an enhanced hexosamine pathway [4]. Diabetic microvascular complications are the primary cause of acquired blindness, nontraumatic lower limb amputation and end-stage renal disease [5]. Timely recognition and management of these complications is mandatory for preventing such damage.

In 1994, the mechanistic (formerly known as mammalian) target of rapamycin (mTOR) was identified as the direct target of rapamycin responsible for its antifungal, immunosuppressive and antitumor effects [6, 7]. Since then, the role of mTOR in cell growth and organismal physiology has been highlighted.

mTOR is a ubiquitous highly conserved serine/threonine protein kinase, that represents the catalytic subunit of protein complexes, mTOR complex 1 (mTORC1) and 2 (mTORC2). mTORC1 plays a fundamental role in maintaining the balance between anabolism and catabolism response to intracellular and environmental stresses via the regulation of diverse processes such as promoting protein, lipid and nucleotide synthesis, and suppressing autophagy [8]. On the other hand, mTORC2 promotes cellular proliferation and survival through several mechanisms, most notably, through the activation of Akt, which is the main effector of insulin/ phosphoinositide 3-kinase (PI3K) signaling [9].

Recently, the role of mTOR in the development of DM and its vascular complications has drawn increased amount of attention. However, its ability to identify diabetic microvascular complications has not been widely studied. The urgent need for a proper marker for microvascular complications targeting early diagnosis and effective management, together with the promising role of mTOR in this field motivated us to investigate the role of mTOR in the detection of diabetic microvascular complications.

## Materials and methods

### Study design and subjects

This case-control study recruited 90 subjects attending the Outpatient Internal Medicine Clinic in Damanhur Teaching Hospital. The study design was approved by the ethics committee of Alexandria University. The participants signed an informed consent form before any study-related procedures took place. The study followed the criteria established by the Declaration of Helsinki.

The study participants were divided into 3 groups.

Group I: Twenty healthy subjects as the control group,

Group II: included 20 subjects with T2DM without complications.

Group III: included 50 subjects with T2DM with microvascular complications.

The following patients were excluded from the study: Patients with type 1 diabetes, recent acute infection within 2 months, severe hepatic impairment, endocrine and metabolic diseases other than T2DM, or a history of malignant disease, pregnant and lactating females.

### Study methods

The following procedures were performed for all the subjects:

#### Clinical assessment

A thorough history was obtained focusing on the medical history, duration of diabetes and associated comorbidities. BMI was calculated (weight in kg divided by height in m2). Waist circumference (WC) and hip circumference were assessed, and the waist to hip ratio (W/H) was calculated as the ratio between the waist measurement and the hip measurement.

Neurological examination was performed including assessment of ankle reflexes and knee reflexes, monofilament assessment, and a test for vibration perception threshold (VPT). Neuropathy was defined if there was a positive monofilament test plus either an absent ankle reflex or a VPT > 25 V.

#### Assessment of the ankle-brachial index (ABI)

A handheld Doppler was applied to assess both the dorsalis pedis and posterior tibial arteries. The systolic pressure of the PT and DP arteries of each leg was measured using a 5 MHz Doppler probe (Nicolet Elite 200 R, VIASYS Healthcare Inc., Madison, WI, USA). A lower value of the two calculated ABI in both limbs was used for statistical analysis [10].

#### Biochemical analysis

Venous blood samples (10 ml from each patient) were collected from the antecubital vein into vacuum tubes with the appropriate additives. Subjects were advised to fast overnight for 12 h and were free of smoking or strenuous exercise.

The withdrawn venous samples were split into 2 parts. The first part was further subdivided into 2 vacuum tubes, a plain tube left to clot at 37℃ to allow subsequent serum separation by centrifugation for immediate assessment of fasting plasma glucose (FPG), serum creatinine and insulin levels, and a dipotassium ethelene diamine tetra-acetic acid (EDTA)-containing vacutainer tube for measuring glycated hemoglobin (HbA1c). The second part of the sample was preserved at -70℃ for the mTOR assay. An enzyme linked immunosorbent assay (ELISA) was used to measure the serum mTOR concentration [11].

Insulin resistance was estimated by the Homeostasis Model Assessment 2 (HOMA2) calculator (HOMA2-IR) using the Oxford online HOMA IR calculator 2008 [12].

A morning urine sample was used to assess the urine albumin/creatinine ratio (ACR), which was repeated twice [13]. In addition, the estimated glomerular filtration rate (eGFR) was calculated using the CKD Epidemiology Collaboration (CKD-EPI) formula [14]. Diabetic kidney disease (DKD) was defined as albuminuria (UACR > 30 mg/g) and/or decreased eGFR < 60 mL/min/1.73 m2.

#### Fundus examination

Fundus examination was performed by an experienced ophthalmologist for the detection of diabetic retinopathy (DR). Fluorescein angiography was ordered once indicated [15].

### Statistical analysis

All the statistical analyses were performed using SPSS software (version 20.0; IBM Corporation, Armonk, NY, USA). The data are presented as the mean ± SD.

For comparing parameters between the three groups, chi square tests and ANOVA were utilized. The Kruskal- Wallis test was applied for non-normally distributed quantitative variables. A *P* value ≤ 0.05 was considered to indicate statistical significance.

To test the relationships between mTOR and different parameters, linear regression analyses were implemented. The independent correlations related to microvascular complications were assessed by multivariate analysis. The utility of the mTOR concentration as a predictor of microvascular complications in patients with T2DM was further evaluated using a receiver operating characteristic (ROC) curve.

## Results

The baseline demographic data are shown in Table 1, where no significant differences were found between the active and control groups regarding sex, age, body mass index, waist circumference or waist-to- hip ratio.

**Table 1:** Comparison of the three studied groups according to baseline characteristics

	Group I (*n* = 20)	Group II (*n* = 20)	Group III (*n* = 50)	Test of Sig.	*p*
**Sex**					
Male	9(45.0%)	6(30.0%)	13(26.0%)	χ2 = 2.421	0.298
Female	11(55.0%)	14(70.0%)	37(74.0%)		
**Age** (years)					
Median (Min. – Max.)	52.0(34.0–66.0)	54.5(30.0–67.0)	56.5(35.0–68.0)	F = 2.700	0.073
Mean ± SD.	51.70 ± 9.57	51.70 ± 9.35	55.66 ± 6.72		
**Weight** (kg)					
Median (Min. – Max.)	90.0(51.0–139.0)	90.5(70.0–120.0)	86.0(55.0–120.0)	F = 0.221	0.802
Mean ± SD.	88.80 ± 19.52	89.95 ± 13.43	87.30 ± 14.85		
**Height** (cm)					
Median (Min. – Max.)	166.0(155.0–182.0)	168.5(155.0–175.0)	165.0(155.0–178.0)	F = 0.214	0.808
Mean ± SD.	165.7 ± 8.08	166.8 ± 5.34	165.9 ± 5.09		
**BMI** (kg/m2)					
Median (Min. – Max.)	32.2(19.9–54.3)	31.5(24.2–43.0)	31.2(20.8–46.9)	F = 0.112	0.894
Mean ± SD.	32.43 ± 7.27	32.22 ± 4.57	31.78 ± 5.28		
**Waist circumference** (cm)					
Median (Min. – Max.)	86.0(68.0–117.0)	84.0(70.0–109.0)	82.0(69.0–112.0)	F = 0.840	0.435
Mean ± SD.	87.50 ± 12.91	84.75 ± 9.97	84.18 ± 8.09		
**Waist/hip ratio**					
Median (Min. – Max.)	0.83(0.67–1.0)	0.82(0.69–0.98)	0.81(0.69–0.99)	F = 2.904	0.060
Mean ± SD.	0.84 ± 0.08	0.83 ± 0.07	0.80 ± 0.06		

χ2: Chi-square test

F: F for ANOVA test

*p*: *p*-value for comparison between the three studied groups

Group I:20 healthy subjects as the control group

Group II:20 subjects with type 2 diabetes without microvascular complications

Group III:50 subjects with type 2 diabetes with microvascular complications

Assessment of the mTOR level revealed a significantly greater level in patients with diabetes who have microvascular complications than in those without microvascular complications and in the control group. These findings, together with other biochemical and clinical parameters, are shown in Tables 2 and 3.

**Table 2:** Comparison of biochemical and clinical parameters among the three studied groups

	Group I (*n* = 20)	Group II (*n* = 20)	Group III (*n* = 50)	F	*p*
**FPG** (mg/dl)					
Median (Min. – Max.)	94.0(86.0–105.0)	138.0(85.0–270.0)	170.0(79.0–430.0)	F = 16.286^*^	< 0.001^*^
Mean ± SD.	94.25^c^ ± 5.48	146.3^b^ ± 46.03	192.3^a^ ± 83.19		
**HbA1C** (%)					
Median (Min. – Max.)	5.2(4.8–5.7)	7.0(6.0–10.0)	8.9(6.3–12.7)	F = 57.192^*^	< 0.001^*^
Mean ± SD.	5.22^c^ ± 0.26	7.39^b^ ± 1.12	8.86^a^ ± 1.57		
**HOMA IR2**					
Median (Min. – Max.)	1.9(0.8–4.7)	2.7(1.5–3.9)	2.2(0.8–15.0)	H = 6.400^*^	0.041^*^
Mean ± SD.	2.08^b^ ± 1.01	2.70^a^ ± 0.64	3.15^ab^ ± 2.91		
**mTOR (ng/ml)**					
Median (Min. – Max.)	3.85(1.70–5.40)	6.95(5.10–10.0)	11.25(8.50–22.0)	F = 88.148^*^	< 0.001^*^
Mean ± SD.	3.86^c^ ± 1.01	6.86^b^ ± 1.14	12.05^a^ ± 3.16		
**ACR (mg/mmol)**					
Median (Min. – Max.)	12.0(5.0–28.0)	22.0(6.0–29.0)	126.5(39.0–1120.0)	H = 67.059^*^	< 0.001^*^
Mean ± SD.	14.40^b^ ± 6.98	19.75^b^ ± 7.04	216.1^a^ ± 260.05		
**eGFR(ml/min/1.73m2)**					
Median (Min. – Max.)	93.5(60.0–137.0)	80.5(60.0–100.0)	60.0(29.0–80.0)	F = 57.937^*^	< 0.001^*^
Mean ± SD.	94.50^a^ ± 18.70	78.90^b^ ± 12.80	57.96^c^ ± 10.93		
**Retinopathy**	0^a^(0.0%)	0^a^(0.0%)	14^b^(28.0%)	13.327^*^	^MC^*p*=0.001^*^
**Nephropathy**	0^a^(0.0%)	0^a^(0.0%)	50^b^(100.0%)	90.00^*^	< 0.001^*^
**Neuropathy**	0^a^(0.0%)	0^a^(0.0%)	12^b^(24.0%)	10.679^*^	^MC^*p*=0.003^*^

**F**: **F for ANOVA test**, pairwise comparison bet. each 2 groups were done using **Post Hoc Test (Tukey)**

H: H for **Kruskal Wallis test**, pairwise comparison bet. each 2 groups were done using **Post Hoc Test (Dunn’s for multiple comparisons test)**

*p*: *p* value for comparison between three studied groups

*: Statistically significant at *p* ≤ 0.05

Means with **Common letters** are not significant (i.e., Means with **Different letters** are significant)

**Group I**: 20 **healthy** subjects as the **control** group

**Group II**: 20 subjects with type 2 **diabetes without complications**

**Group III**: 50 subjects with type 2 **diabetes with microvascular complications**

**Table 3:** Relation between mTOR and different parameters in Group II and III

	No.	mTOR			*t*	*p*
		Min. – Max.	Mean ± SD.	Median		
**Nephropathy**						
No	**20**	5.10–10.0	6.86 ± 1.14	6.95	10.078^*^	< 0.001^*^
Yes	**50**	8.50–22.0	12.05 ± 3.16	11.25		
**Neuropathy**						
No	**58**	5.10–22.0	9.81 ± 3.36	9.45	4.313^*^	< 0.001^*^
Yes	**12**	9.50–18.20	14.22 ± 2.38	14.40		
**Retinopathy**						
No	**56**	5.10–13.80	9.20 ± 2.21	9.35	9.740	< 0.001^*^
Yes	**14**	12.30–22.0	16.04 ± 2.88	15.15		

***t***: **student*t*-test**

*p*: *p*-value for comparing between the studied categories

*: Statistically significant at *p* ≤ 0.05

Correlation between mTOR and different parameters in the whole population showed positive significant correlation between mTOR and FPG (*r* = 0.508, *P* < 0.001), HbA1C (*r* = 0.530, *P* < 0.001) and HOMA IR2 (*r* = 0.559, *P* < 0.001). On the other hand, there was negative significant correlation between mTOR and eGFR (*r*=-0.370, *P* = 0.002). The details are shown in Table 4.

**Table 4:** Correlations between mTOR levels and different parameters in each group

	mTOR							
	Group I (Control) (*n* = 20)		Group II (*n* = 20)		Group III (*n* = 50)		Total cases (*n* = 70)	
	*r*	*p*	*R*	*p*	*r*	*P*	*r*	*p*
ABPI	0.058	0.808	0.092	0.701	-0.094	0.518	-0.135	0.265
FPG	-0.047	0.845	0.242	0.305	0.473^*^	0.001^*^	0.508^*^	< 0.001^*^
HbA1C	0.200	0.398	0.435	0.055	0.373^*^	0.008^*^	0.530^*^	< 0.001^*^
HOMA IR2	0.172	0.469	0.429	0.059	0.679^*^	< 0.001^*^	0.559^*^	< 0.001^*^
ACR	0.260	0.269	0.314	0.178	-0.055	0.703	0.210	0.081
eGFR	0.183	0.440	0.063	0.792	0.098	0.498	-0.370^*^	0.002^*^
BMI	0.147	0.535	0.008	0.973	-0.170	0.237	-0.136	0.261
Weight	0.309	0.185	-0.133	0.575	-0.093	0.522	-0.125	0.303

**r: Pearson coefficient**

*: Statistically significant at *p* ≤ 0.05

**Group I**: 20 **healthy** subjects as the **control** group

**Group II**: 20 subjects with type 2 **diabetes without complications**

**Group III**: 50 subjects with type 2 **diabetes with microvascular complications**

**Fig. 1:**
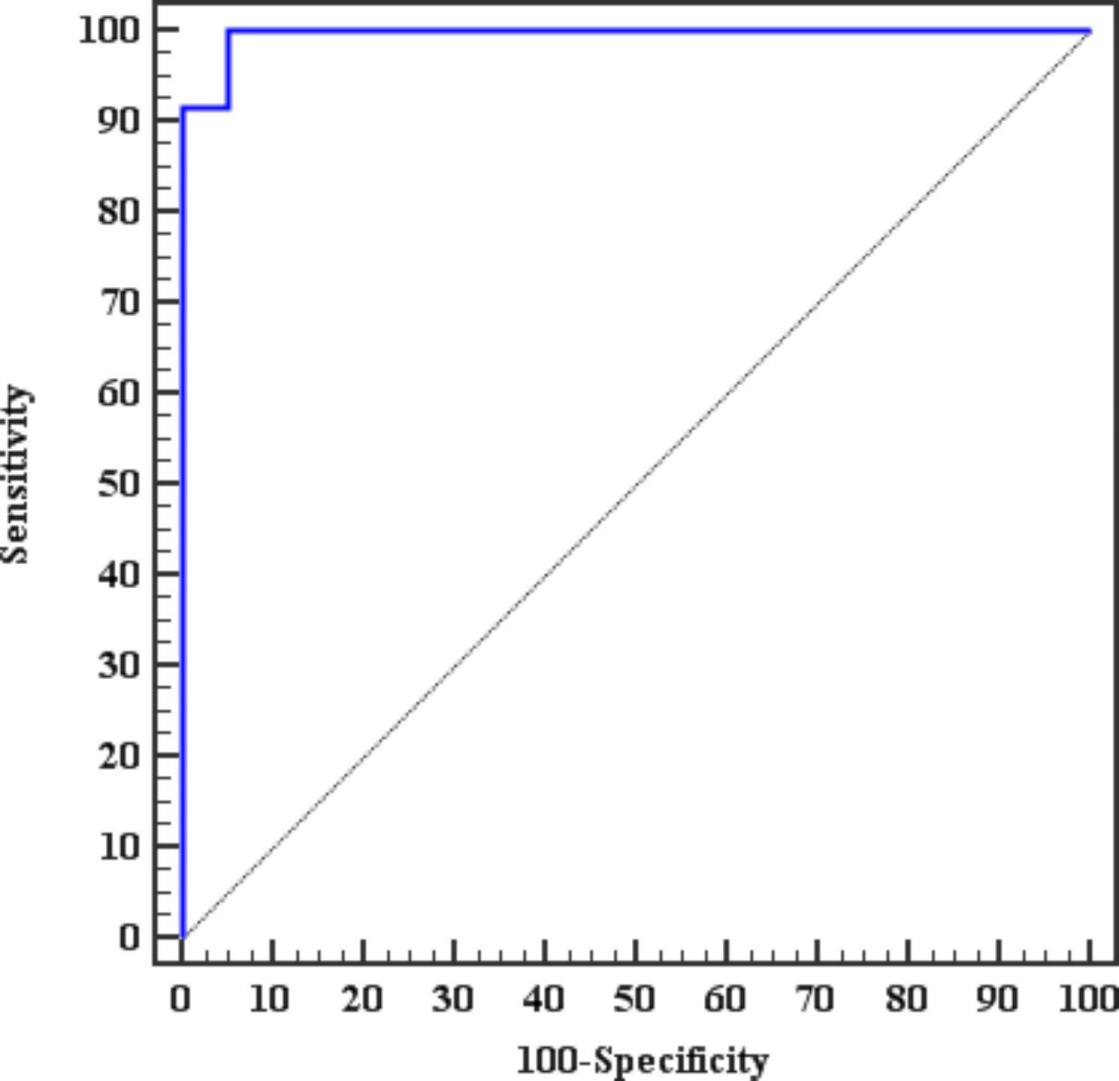
ROC curve for the ability of the mTOR to predict microvascular complications in patients with diabetes

Multivariate analysis of the parameters associated with the occurrence of microvascular complications in subjects with diabetes showed that the mTOR and HbA1c values were independently associated factors (Table 5). The ROC curve showed an mTOR cutoff value of 8 ng/ml to predicting patients with microvascular complications with a sensitivity of 100% and specificity of 95% with an AUC of 0.983 and a *p*-value < 0.001. Figure 1.

**Table 5:** Univariate and multivariate analyses of the parameters affecting the occurrence of microvascular complications (*n* = 70 diabetes cases)

	Univariate		^#^Multivariate	
	*p*	OR (95%C.I)	*p*	OR (95%C.I)
Sex/female	0.734	1.220(0.388–3.838)		
Age	0.060	1.068(0.997–1.144)		
Weight	0.485	0.987(0.952–1.024)		
Height	0.525	0.967(0.873–1.072)		
BMI	0.737	0.983(0.887–1.089)		
Waist circumference	0.801	0.992(0.935–1.053)		
Waist/hip ratio	0.160	0.002(0.0–10.951)		
FPG	0.032^*^	1.011(1.001–1.021)	0.187	0.910(0.790–1.047)
HOMA IR2	0.498	1.097(0.840–1.432)		
mTOR	0.001^*^	17.736(3.041–103.433)	0.010^*^	25.997(2.201–307.116)
HbA1c	0.001^*^	2.274(1.378–3.753)	0.015^*^	3.602(1.921–3.964)

OR: odds ratio, C.I: Confidence interval

#: All variables with *p* < 0.05 were included in the multivariate analysis

*: Statistically significant at *p* ≤ 0.05

## Discussion

mTOR is considered a prime regulator of cellular growth and metabolism in reaction to several factors such as growth factors, nutrients, and several extracellular signals. mTOR has been the focus of previous research in the field of neoplastic disorders in several studies. However, recently the dysregulation of mTOR has been related to several metabolic conditions, notably obesity and T2DM [16].

Several in vivo physiological studies have shown that the mTORC1 pathway is essential for glucose homeostasis at the organismal level and have confirmed its important function in maintaining the balance of cellular metabolism, as revealed by Kennedy & Lamming. Nevertheless, exaggerated activation of mTORC1 has also been linked to metabolic derangements; thus tight balancing of mTORC1 in response to different metabolic stimuli is critical in metabolically active tissues exposed to variable modifications [17].

In this study, mTOR was significantly positively correlated with various parameters of the glycemic profile including FPG, and HbA1c. Inconsistent with our work, this was explained previously by Eisenreich et al., who reported that hyperglycemia and related activated growth factors, result in the activation of mTOR predominantly through the phosphatidylinositol 3-kinase/Akt signaling pathway. The activated growth factors included insulin-like growth factor, platelet-derived growth factor, and epidermal growth factor [18].

The role of mTOR in glucose homeostasis seems to be intricate, with some opposing effects depending on the duration and level of mTOR activation. This relationship presumably follows a U-shaped curve, such that both increased and decreased mTOR activity have unfavorable impacts on metabolism [19]. Fang et al. [20] demonstrated that short-term rapamycin treatment had detrimental metabolic effects on mice. However, prolonged treatment with rapamycin was associated with improved metabolic status and enhanced insulin sensitivity. This could partially explain the ‘‘Janus effect’’ of mTOR inhibitors on glucose homeostasis [21].

Insulin resistance is a cornerstone of the pathogenesis of T2DM. The association between insulin resistance and cardio-metabolic risk is well established. Despite the great variability in the threshold values, HOMA-IR has been used to define insulin resistance. In the present study, mTOR was significantly positively correlated with HOMA IR2 [22].

mTORC1 activation enhances insulin resistance in the main insulin-target organs. In adipose tissue, mTORC1 inhibits insulin signaling via ribosomal S6 kinase 1 (S6K1), which occurs due to the serine-phosphorylation of insulin receptor substrate-1 (IRS-1) [23]. This phenomenon was also reported in the liver and skeletal muscles of obese rats [24]. In hepatocytes, the mTOR/S6K1-mediated serine phosphorylation of IRS-1 promotes gluconeogenesis through impairment of the PI3K-Akt metabolic pathway. Additionally, proteasomal degradation of insulin receptor substrate- 2 (IRS-2) is enhanced through a mechanism similar to that of mTOR/S6K1-mediated serine phosphorylation [25]. In muscles, in addition to IRS-1 phosphorylation, mTORC1 reduces muscle mass and oxidative function [26].

On the other hand, mTORC1 is considered a positive regulator of β-cell mass and function; thus, mTOR activation leads initially to improved insulin secretion. Nevertheless, sustained mTOR activation leads to β-cell exhaustion, reduced cell survival and enhanced apoptosis, and eventually deterioration of insulin secretion capacity [27].

The results of this study showed a significantly higher level of mTOR in diabetic subjects with microvascular complications than in diabetic subjects without complications, and in the control group.

Our results showed a significant negative correlation between mTOR levels and the eGFR. Moreover, when subjects with complications were further classified based on their eGFR and/ or the presence of albuminuria, the mTOR level was significantly greater in those with DKD.

The classic presentation of kidney disease in patients with diabetes, known as diabetic kidney disease, is characterized by the detachment of podocytes from the epithelial basement membrane in the glomerulus. The detachment of glomerular podocytes is followed by their loss and subsequent cellular loss of proximal tubules, and consequently albuminuria develops. The critical role of insulin-activated mTORC1 in the development and progression of DKD has been previously studied. In agreement with our results, mTOR has been demonstrated previously to be involved in hyperglycemia-induced renal diseases [28].

Additionally, several pathways have been suggested previously for the early prevention of DKD focusing on the pivotal role of mTOR through rapamycin treatment [28] or reducing the number of mTORC1 copies in podocytes [29, 30].

In vitro, Lu et al. reported that pretreatment of glomerular mesangial cells with rapamycin mitigated oxidative stress and decreased the number of apoptotic cells, which were induced by high glucose concentrations and resulted in the downstream effects of mTOR activation. Furthermore, in vivo, rapamycin treatment in diabetic rats attenuated albuminuria and improved renal function in diabetic rats [31]. Similarly, Wu et al. [32] reported that early glomerular pathological changes (mesangial expansion, glomerular hypertrophy and basement membrane thickening) were attenuated, both in vivo and in vitro, by inhibiting Akt/mTOR/p70S6K signaling activity.

In addition to the aforementioned effects of mTOR inhibition, Yasuda et al. [33] showed that mTOR inhibition ameliorated podocyte apoptosis. The inhibition of podocyte apoptosis in DKD is considered a fundamental therapeutic target; owing to the importance of podocytes in maintaining the integrity of the glomerular filtration barrier and because podocytes are terminally differentiated cells that are unable to proliferate.

Earlier this year, Dong and colleagues demonstrated that the application of rapamycin, in high glucose-induced human renal glomerular endothelial cells could significantly increase platelet and endothelial cell adhesion molecule-1 (CD31) and vascular endothelial-cadherin expression, while reversing the over-expression of Collagen 1 and α-smooth muscle actin and alleviating endothelial-to-mesenchymal transition (EndMT), which plays a key role in the development of DKD [34].

Consistent with our findings, mTORC1 has been implicated in the development of diabetic retinopathy. Diabetic retinopathy is a complex and multifactorial process. Exposure to sustained hyperglycemia leads to the activation of oxidative stress and the overproduction of reactive oxygen species (ROS) [35]. This is followed by a significant increase in inflammatory cytokines and hypoxia-stimulated vascular endothelial growth factor (VEGF) production. VEGF stimulates retinal angiogenesis or neovascularization, promoting further development of diabetic retinopathy.

Previous studies have shown that the insulin/mTOR pathway stimulates the production of VEGF in the retinal pigment epithelial cell (RPE). Insulin and insulin-like growth factor-1 (IGF-1) are involved in angiogenesis and DR. These findings explain the observations relating intensified insulin treatment to the worsening of diabetic retinopathy [36]. Moreover, suppression of DR progression in insulin-treated mice was observed following berberine treatment, chiefly through attenuation of AKT/mTOR-mediated retinal expression of hypoxia-inducible factor-1α (HIF-1α) and VEGF [37].

Furthermore, PI3K/AKT/mTOR activation promotes angiogenesis through the interaction between Akt and Ras homolog gene family member B (RhoB). This interaction enhances the development and survival of retinal endothelial cells during the process of vascular genesis [38, 39].

Additionally, He et al. [40] revealed that PI3K/AKT/mTOR activation was associated with endothelial-mesenchymal transition in streptozotocin rats with DR. In addition, maternally expressed gene 3 (MEG3) overexpression led to EndMT suppression through inhibition of the PI3K/AKT/mTOR pathway.

Diabetic peripheral neuropathy is the most common chronic complication among patients with T2DM. Hyperactive mTORC1 is reported to induce chronic neuropathic changes by interfering with synaptic integrity. Moreover, the suppression of mTORC1 activity may result in antinociceptive effects in experimental models of inflammatory and neuropathic pain [41, 42]. mTOR is present in the sensory nervous system and its downstream signals contribute to transmission, modulation, and development of peripheral pain sensitization [42, 43].

Liu and colleagues demonstrated that impaired autophagy due to enhanced activity of the PI3K/AKT/mTOR pathway was associated with significantly diminished paw mechanical withdrawal thresholds (MWTs) in T2DM rat models. Moreover, following PI3K inhibitor administration, the MWT was significantly improved, and this change was accompanied by suppression of the PI3K/AKT/mTOR pathway. Thus, hyperalgesia in diabetic rats was alleviated by inhibition of the PI3K/AKT/mTOR signaling pathway [44].

In contrast, Dong et al. reported that high glucose (HG) treatment of neuronal Schwann cells leads to enhanced apoptosis and reduced cell proliferation as a result of downregulated Akt/mTOR pathway. These effects were reversed by Muscone through the activation of the Akt/mTOR signaling pathway [45].

Similarly, Zhang et al. recently illustrated that artesunate alleviated nerve injury induced by hyperglycemia, both in vivo and in vitro, by inhibiting apoptosis and enhancing Schwann cell viability via activation of the PI3K/AKT/mTOR signaling pathway [46].

These contradictory results emphasize the fine balance required for mTOR and related pathway activation to maintain neuronal cell integrity and function by preserving the delicate interaction between cell survival and cell death as modulated by apoptotic and autophagic pathways [47].

The results of this study point toward the role of mTOR as a predictor of the occurrence of diabetes-related microvascular complications in the future. The ROC curve showed an mTOR cutoff value of 8 ng/ml for predicting patients with microvascular complications with a sensitivity of 100% and specificity 95% with an AUC of 0.983 and a *p*-value < 0.001.

This study had some limitations; including that it was a single-center study that included only Egyptian subjects. However, further investigations are needed to confirm the application of these findings to other populations. Future research is needed to confirm whether mTOR levels are independently associated with the development of microvascular complications. In addition, the size of the studied sample was small.

## Conclusion

In this case- control study, mTOR was able to identify microvascular complications in T2DM patients with excellent diagnostic performance, suggesting that mTOR is a promising predictor of diabetes-related microvascular complications in patients with T2DM.

## Data Availability

No datasets were generated or analysed during the current study.
